# Excessive Daytime Sleepiness, but Not Insomnia Is Associated With Dyslipidaemia in Patients With Obstructive Sleep Apnoea Participating in ESADA

**DOI:** 10.1111/jsr.70240

**Published:** 2025-11-11

**Authors:** Andras Bikov, Sebastien Bailly, Ulla Anttalainen, Tarja Saaresranta, Ozen K. Basoglu, Sophia Schiza, Izolde Bouloukaki, Pawel Sliwinski, Athanasia Pataka, Dries Testelmans, Francesco Fanfulla, Haralampos Gouveris, Ludger Grote, Stefan Mihaicuta, the ESADA collaborators, P. Steiropoulos, J. Verbraecken, E. Petiet, Georgia Trakada, I. Fietze, T. Penzel, Ondrej Ludka, I. Bouloukaki, S. Schiza, W. T. McNicholas, S. Ryan, R. L. Riha, J. A. Kvamme, L. Grote, J. Hedner, D. Zou, Katrien Hertegonne, Dirk Pevernagie, S. Bailly, J. L. Pépin, R. Tamisier, H. Hein, O. K. Basoglu, M. S. Tasbakan, P. Joppa, R. Staats, Dries Testelmans, Alexandros Kalkanis, Haralampos Gouveris, K. Ludwig, C. Lombardi, G. Parati, M. R. Bonsignore, Francesco Fanfulla, M. Petitjean, G. Roisman, M. Drummond, M. van Zeller, W. Randerath, S. Mathes, Z. Dogas, T. Galic, A. Pataka, S. Mihaicuta, U. Anttalainen, T. Saaresranta, P. Sliwinski

**Affiliations:** ^1^ Wythenshawe Hospital Manchester University NHS Foundation Trust, Manchester Academic Health Science Centre Manchester UK; ^2^ Division of Infection, Immunity & Respiratory Medicine, Faculty of Biology, Medicine and Health The University of Manchester Manchester UK; ^3^ Grenoble Alpes University Inserm, CHU Grenoble Alpes Grenoble France; ^4^ Division of Medicine, Department of Pulmonary Diseases Turku University Hospital and Sleep Research Centre Turku Finland; ^5^ Department of Pulmonary Diseases and Clinical Allergology University of Turku Turku Finland; ^6^ Department of Respiratory Medicine, Faculty of Medicine Ege University Izmir Turkey; ^7^ Sleep Disorders Center, Department of Respiratory Medicine, School of Medicine University of Crete Heraklion Greece; ^8^ Second Department of Respiratory Medicine Institute of Tuberculosis and Lung Diseases Warsaw Poland; ^9^ Respiratory Failure Unit, G Papanikolaou Hospital, School of Medicine Aristotle University of Thessaloniki Thessaloniki Greece; ^10^ Department of Pneumology University Hospitals Leuven Leuven Belgium; ^11^ Department of Chronic Diseases and Metabolism Laboratory of Respiratory Diseases and Thoracic Surgery (BREATHE) Leuven Belgium; ^12^ Sleep Medicine Unit—Istituti Clinici Scientifici Maugeri—Istituto Scientifico di Pavia e Montescano IRCCS Pavia Italy; ^13^ ENT Department at Mainz University Hospital Mainz Germany; ^14^ Center for Sleep and Wake Disorders, Institute of Medicine, Sahlgrenska Academy University of Gothenburg Gothenburg Sweden; ^15^ Pulmonary Medicine, Sahlgrenska University Hospital Gothenburg Sweden; ^16^ Center for Research and Innovation in Precision Medicine of Respiratory Diseases, Department of Pulmonology “Victor Babes” University of Medicine and Pharmacy Timisoara Timisoara Romania

**Keywords:** apnoea, cardiovascular disease, insomnia, lipids, sleep, sleepiness

## Abstract

Excessive daytime sleepiness (EDS) as well as insomnia have been associated with a higher risk for cardiovascular disease in patients with obstructive sleep apnoea (OSA). The link is not fully understood but may involve dyslipidaemia. The aim of the study was to analyse if the EDS and insomnia phenotypes were associated with deranged serum lipid values in patients with OSA recruited from a European real‐world cohort. Patients with OSA and a full lipid profile participating in the ESADA database were analysed (*n* = 12,153). Based on their symptoms, they were categorised into EDS (*n* = 3123), EDS + insomnia (*n* = 2091), insomnia (*n* = 2862) and non‐EDS non‐insomnia (*n* = 4077) subgroups. Nonparametric ANCOVA adjusted for age, body mass index, smoking, alcohol, study site, apnoea‐hypopnoea index and time spent with saturation below 90%, followed by Dunn's test and Bonferroni correction, was used to compare lipid values between the groups. The analyses were also performed in predefined subgroups. There were significant differences in total cholesterol (TC), LDL‐cholesterol (LDL‐C), HDL‐cholesterol (HDL‐C) and triglyceride (TG) values between the four groups (all *p* < 0.01). Patients with EDS had the highest TC (5.11 ± 1.08 vs. 5.00 ± 1.10, 5.03 ± 1.12, 5.04 ± 1.10 mmol/L, EDS vs. EDS + insomnia, insomnia, non‐EDS non‐insomnia, respectively), LDL‐C (3.12 ± 0.97 vs. 3.01 ± 0.98, 3.02 ± 1.00, 3.09 ± 0.98 mmol/L) and TG (1.86 ± 1.04 vs. 1.76 ± 0.97, 1.69 ± 0.90, 1.75 ± 0.93 mmol/L) values and the lowest HDL‐C results (1.18 ± 0.33 vs. 1.21 ± 0.34, 1.26 ± 0.38, 1.20 ± 0.34). Interestingly, patients with insomnia had the highest HDL‐C values. EDS is significantly associated with dyslipidaemia in patients with OSA. Further studies are warranted to understand the link in detail and to translate it into clinical practice.

## 1 Introduction

Obstructive sleep apnoea (OSA) is a common multifactorial disease characterised by recurrent collapse of the upper airways during sleep resulting in chronic intermittent hypoxia and sleep fragmentation. Although OSA is strongly associated with cardiovascular disease (CVD) morbidity and mortality (Redline et al. 2023), it is not fully clear which patient is at the highest risk for CVD. Both symptoms of insomnia (Lechat et al. 2022a) and excessive daytime sleepiness (EDS) (Mazzotti et al. 2019) were associated with increased CVD risk in the Sleep Heart Health Study (SHHS); however, the reason for the association between symptoms and CVD has not been explored. Importantly, the severity of insomnia in patients with OSA can lead to more significant EDS (Gouveris et al. 2025) suggesting that these symptoms should not be investigated separately.

Dyslipidaemia is a mainstay component of atherosclerosis and consequential CVD (Raggi et al. 2018) and lipid values are routinely used in clinical practice to predict future CVD risk (Miller et al. 2011; Wilson et al. 1998). Not surprisingly, dyslipidaemia plays an essential role in OSA‐associated heightened CVD risk (Meszaros and Bikov 2022). Analysing the European Sleep Apnoea Database (ESADA), an independent relationship has previously been reported between OSA and higher total cholesterol (TC), low‐density lipoprotein cholesterol (LDL‐C) and triglyceride (TG) and lower high‐density lipoprotein cholesterol (HDL‐C) levels (Gündüz et al. 2018). In line with this, investigation of dyslipidaemia can shed light on the nature of the relationship between symptoms‐based OSA phenotypes and CVD risk.

EDS was related to higher levels of TG and lower levels of HDL‐C in patients with severe OSA (Huang et al. 2016), but the results were not replicated by other studies (Bonsignore et al. 2012; Li et al. 2019; Nena et al. 2012). The lack of relationship is surprising considering that insulin resistance (Chen et al. 2024) and a higher burden of appetite‐promoting hormones (Sánchez‐de‐la‐Torre et al. 2011) were associated with EDS in patients with OSA. Importantly, EDS is often evaluated by subjective scores, such as the Epworth Sleepiness Scale (ESS) which shows significant interpopulation variability (Bonsignore et al. 2021) that can itself explain the discrepancy between studies.

Insomnia was associated with dyslipidaemia in large population‐based cohort studies (Lee et al. 2024; Syauqy et al. 2019; Wang et al. 2023). The reason for the relationship is not fully understood, but short sleep duration (Deng et al. 2017; Fernandez‐Mendoza et al. 2017) and associated higher calorie intake (Wrzosek et al. 2018) may play a role. In addition, a genetic link between insomnia and higher TG and lower HDL‐C was reported analysing the UK Biobank cohort (Liu et al. 2021). Interestingly, according to the National Health and Nutrition Examination Surveys study, the link existed only in patients taking hypnotics (Vozoris 2016). It is important to note that insomnia symptoms are often reported by patients with delayed phase sleep disorder, and dyslipidaemia may be associated with circadian misalignment rather than chronic insomnia (Csoma and Bikov 2023). Furthermore, periodic limb movements in sleep may also contribute to insomnia symptoms as well as dyslipidaemia in patients with OSA (Bikov et al. 2024). Finally, insomnia may trigger anxiety, which was reported to mediate the association between insomnia and higher TG levels (Hsu and Chang 2022). Of note, the definition of insomnia was different in the studies potentially contributing to conflicting results (Lee et al. 2024; Syauqy et al. 2019; Wang et al. 2023).

Similarly to the variability in symptoms, lipid values also show high interindividual variability in OSA depending on sex, age, genetic, clinical and lifestyle factors (Bikov et al. 2020; Meszaros et al. 2020). Hence, data on specific cohorts analysing dyslipidaemia in OSA cannot be fully interpolated to other populations. Large multicentre studies are therefore warranted to better understand the relationship between symptoms and dyslipidaemia in OSA. The ESADA is a multicentre collaborative network which at the time of our analysis included more than thirty‐seven thousand patients referred for assessment with symptoms suggestive of OSA (Hedner et al. 2011). As part of their assessment, many subjects had fasting blood samples, including lipid profiles. The impact of insomnia and EDS on CVD was analysed by two reports in ESADA (Anttalainen et al. 2019; Saaresranta et al. 2016). In these studies patients with insomnia had a higher prevalence of CVD, suggesting that dyslipidaemia may play a role. Interestingly, while the combination of EDS and insomnia symptoms increased the prevalence of metabolic disease, there was no further effect on CVD suggesting that metabolic pathways may be differently affected by EDS and insomnia (Anttalainen et al. 2019).

The aim of this study was to analyse lipid values in detail, more specifically their relationship with EDS and insomnia in patients participating in the ESADA.

## 2 Methods

### 2.1 Study Design and Subjects

**FIGURE 1:**
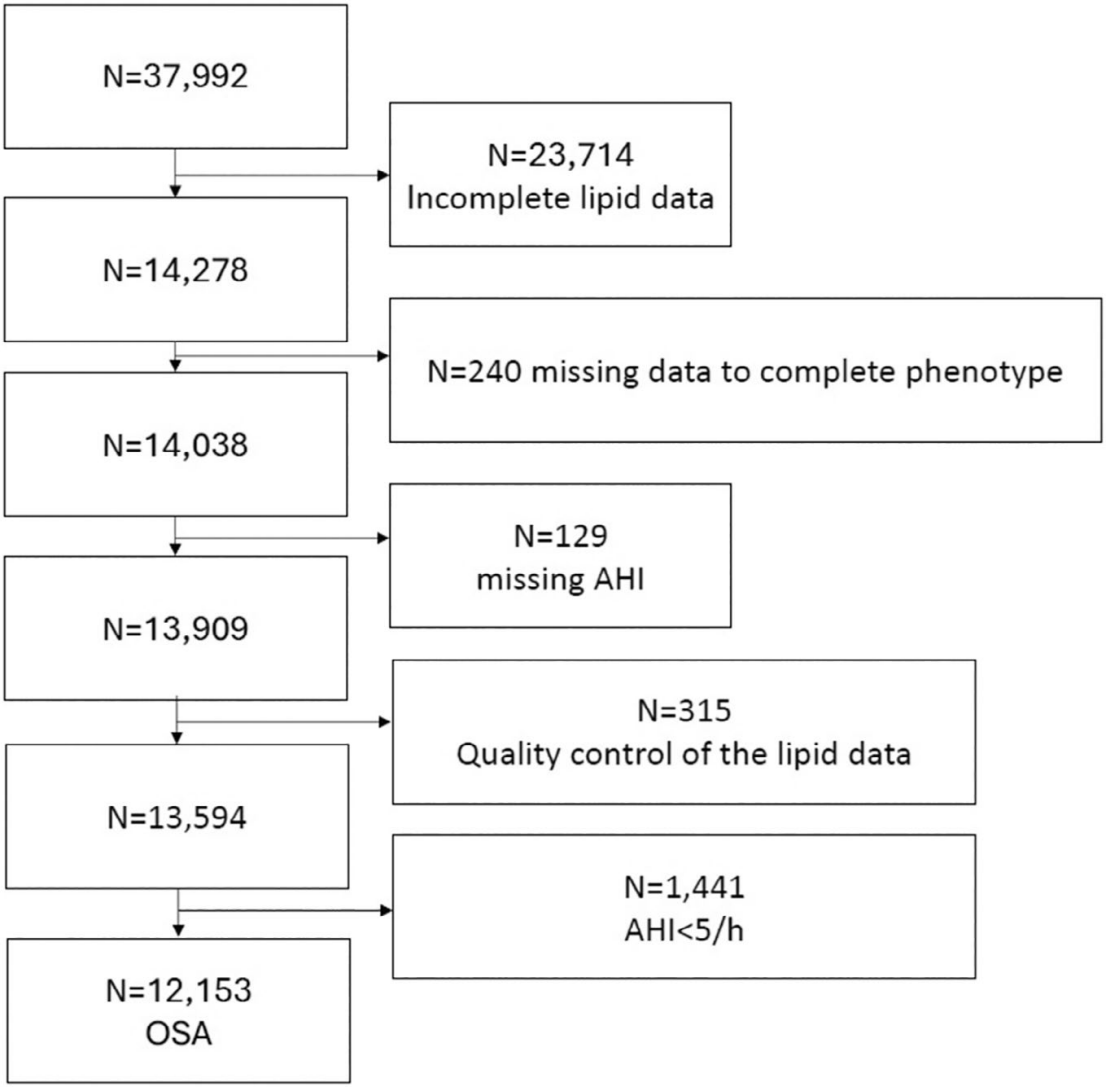
Flowchart for patient selection. AHI, apnoea‐hypopnoea index; OSA, obstructive sleep apnoea.

At the time of data extraction (16 October 2023) the ESADA comprised 37,992 participants recruited at 31 sites. We excluded patients with incomplete lipid data and those following quality control of the data (i.e., TC was less than the sum of HDL‐C and LDL‐C), those who had missing information on insomnia phenotype and those who had missing or incomplete sleep data. As a result, 12,153 patients with OSA from 29 centres were analysed (Figure 1).

For this project we captured demographic data, comorbidities, medications, the ESS, lipid profile including TC, LDL‐C, HDL‐C and TG levels and the sleep study data. Patients were categorised into the EDS group if ESS was > 10. The insomnia group included patients with physician‐diagnosed insomnia, subjective sleep latency > 30 min, self‐reported total sleep time < 6 h and/or hypnotic use defined by the ATC code N05 (Saaresranta et al. 2016). The study was approved by individual research ethics committees and all patients gave their informed consent prior to the study.

### 2.2 Sleep Studies

OSA was diagnosed and evaluated with polysomnography in 8696 cases and with cardiorespiratory polygraphy in 3457 patients. The American Academy of Sleep Medicine 2007 criteria were used (Iber 2007) for respiratory scoring. For the analysis, we captured apnoea–hypopnoea index (AHI), oxygen desaturation index (ODI) and the time spent with oxygen saturation below 90% (T90). OSA was diagnosed if the AHI was ≥ 5/h.

### 2.3 Statistical Analysis

The JASP 0.14 (JASP Team, University of Amsterdam, Amsterdam, The Netherlands) software was used for statistical analysis. Demographic and clinical characteristics were compared using ANOVA and Chi‐square tests. Normality was tested with the Kolmogorov–Smirnov test. Due to the nonparametric distribution of lipid values nonparametric ANCOVA adjusted for age, BMI, smoking status, alcohol units, AHI, T90 and the recruiting centre, followed by Dunn's test and Bonferroni correction, was used to compare lipid values between the groups. Comparisons were performed in the whole group, in females and males separately, in patients without lipid lowering medications (defined by the ATC code C10), in patients without hypnotics (defined by the ATC code N05, *n* = 550), in patients without diabetes, in severe OSA subjects participating in ESADA and in patients who had polysomnography as a diagnostic test. To analyse the effect of various determinants of insomnia criterion on lipid values, further ANCOVA tests were performed adjusted for the same confounders. The results are expressed as mean ± standard deviation. A *p* < 0.05 was considered significant.

## 3 Results

### 3.1 Demographics and Clinical Characteristics

Based on their symptoms patients were categorised into EDS (*n* = 3123), EDS + insomnia (*n* = 2091), insomnia (*n* = 2862) and non‐EDS non‐insomnia (*n* = 4077) subgroups. There were significant differences in most of the parameters between the groups. Most importantly, the ratio of females, the prevalence of hypertension, CVDs and diabetes as well as the ratio of subjects taking lipid‐lowering medications, were higher in the two insomnia groups. In contrast, patients complaining of EDS had worse OSA severity and more prolonged overnight hypoxia (Table 1).

**TABLE 1:** Clinical characteristics.

	EDS *n* = 3123	EDS + insomnia *n* = 2091	Insomnia *n* = 2862	Non‐EDS non‐insomnia *n* = 4077	*p*
Age (years)	51 ± 12	54 ± 12	56 ± 13	54 ± 12	< 0.01
Sex (females %)	25.0	30.3	34.1	22.9	< 0.01
BMI (kg/m^2^)	33.4 ± 6.7	34.0 ± 7.1	32.2 ± 6.4	31.8 ± 6.0	< 0.01
Waist circumference (cm)	113 ± 15	114 ± 16	110 ± 15	110 ± 14	< 0.01
Hip circumference (cm)	113 ± 13	115 ± 14	112 ± 13	110 ± 11	< 0.01
Neck circumference (cm)	43 ± 4	42 ± 4	41 ± 4	42 ± 4	< 0.01
Systolic blood pressure (mmHg)	134.3 ± 16.6	133.4 ± 16.8	132.9 ± 16.4	133.5 ± 16.8	0.01
Diastolic blood pressure (mmHg)	80.9 ± 11.6	79.5 ± 11.2	80.0 ± 11.1	80.4 ± 11.1	< 0.01
Hypertension (%)	43.9	48.4	48.8	44.9	< 0.01
Ischaemic heart disease (%)	8.9	10.8	9.5	8.8	0.06
Cerebrovascular disease (%)	1.5	2.8	3.3	1.8	< 0.01
Type II diabetes (%)	17.4	20.4	16.2	14.2	< 0.01
Restless leg syndrome (%)	0.6	1.0	1.1	0.6	0.05
Chronic insomnia (%)	0.0	6.3	9.2	0.0	< 0.01
Alcohol intake (units)	2.2 ± 5.8	2.0 ± 4.5	2.0 ± 5.0	2.4 ± 6.1	0.02
Smokers (%)	28.8	29.1	24.8	24.6	< 0.01
Patients on lipid lowering medications (%)	18.3	26.6	26.2	22.9	< 0.01
Patients on hypnotics (%)	0.0	9.2	11.7	0.0	< 0.01
Prolonged sleep latency (%)	0.0	70.0	76.0	0.0	< 0.01
Short sleep time (%)	0.0	51.8	38.6	0.0	< 0.01
AHI (/hour)	43.0 ± 28.6	41.5 ± 27.2	33.8 ± 23.3	35.9 ± 24.5	< 0.01
ODI (/hour)	39.0 ± 30.3	39.0 ± 29.1	30.4 ± 24.7	31.4 ± 26.0	< 0.01
T90 (minutes)	65.3 ± 94.2	68.2 ± 88.6	46.8 ± 75.3	44.3 ± 75.3	< 0.01
ESS score	14.9 ± 3.2	14.6 ± 3.0	5.8 ± 2.9	6.0 ± 2.8	< 0.01

Abbreviations: AHI, apnoea‐hypopnoea index; BM, body mass index; EDS, excessive daytime sleepiness; ESS, excessive daytime sleepiness; ODI, oxygen desaturation index; T90, time spent with oxygen saturation below 90%.

### 3.2 Comparison of Lipid Results Between the Four Groups

There were significant differences between the four groups in all lipid values. The EDS group had the highest levels of TC and TG and the lowest levels of HDL‐C. The two insomnia groups had the lowest levels of LDL‐C. The non‐EDS insomnia group had the highest concentration of HDL‐C. The lipid results and intergroup comparisons are summarised in Table 2.

**TABLE 2:** Comparison of lipid values between the four groups.

	EDS *n* = 3123	EDS + insomnia *n* = 2091	Insomnia *n* = 2862	Non‐EDS non‐insomnia *n* = 4077	*p*
TC (mmol/L)	5.11 ± 1.08^b^, ^c^, ^d^	5.00 ± 1.10^a^	5.03 ± 1.12^a^	5.04 ± 1.10^a^	< 0.01
LDL‐C (mmol/L)	3.12 ± 0.97^b^, ^c^	3.01 ± 0.98^a^, ^d^	3.02 ± 1.00^a^, ^d^	3.09 ± 0.98^b^, ^c^	< 0.01
HDL‐C (mmol/L)	1.18 ± 0.33^b^, ^c^	1.21 ± 0.34^a^, ^c^, ^d^	1.26 ± 0.38^a^, ^b^, ^d^	1.20 ± 0.34^b^, ^c^	< 0.01
TG (mmol/L)	1.86 ± 1.04^b^, ^c^, ^d^	1.76 ± 0.97^a^	1.69 ± 0.90^a^, ^d^	1.75 ± 0.93^a^, ^c^	< 0.01

Abbreviations: HDL‐C, high‐density lipoprotein cholesterol; LDL‐C, low‐density lipoprotein cholesterol; TC, total cholesterol; TG, triglycerides.

^a^ *p* < 0.05 versus EDS.

^b^ *p* < 0.05 versus EDS + insomnia.

^c^ *p* < 0.05 versus insomnia.

^d^ *p* < 0.05 versus non‐EDS non‐insomnia.

### 3.3 Sex‐Related Differences

In females, there were no differences in TC or TG levels between the groups. The LDL‐C concentrations were lower in the insomnia group than in females without EDS or insomnia. The HDL‐C levels were the highest in the insomnia group.

In contrast, in males, there were differences in all lipid levels between the four groups. Patients with EDS had the highest TC and TG levels. The LDL‐C levels tended to be the lowest; the HDL‐C levels tended to be the highest in the two insomnia groups (Table 3).

**TABLE 3:** Comparison of lipid values between the four groups in females and males.

Females					
	EDS *n* = 781	EDS + insomnia *n* = 633	Insomnia *n* = 976	Non‐EDS non‐insomnia *n* = 933	*p*
TC (mmol/L)	5.26 ± 1.09	5.19 ± 1.10	5.21 ± 1.12	5.27 ± 1.11	0.40
LDL‐C (mmol/L)	3.16 ± 0.98	3.09 ± 1.00	3.07 ± 1.02^d^	3.21 ± 1.03^c^	< 0.01
HDL‐C (mmol/L)	1.35 ± 0.37^c^	1.37 ± 0.38^c^	1.44 ± 0.43^a^, ^b^, ^d^	1.39 ± 0.39^c^	< 0.01
TG (mmol/L)	1.64 ± 0.82	1.64 ± 0.83	1.56 ± 0.75	1.56 ± 0.78	0.19

Males					
	EDS *n* = 2342	EDS + insomnia *n* = 1458	Insomnia *n* = 1886	Non‐EDS non‐insomnia *n* = 3144	*p*
TC (mmol/L)	5.07 ± 1.07^b^, ^c^, ^d^	4.92 ± 1.09^a^	4.94 ± 1.11^a^	4.98 ± 1.09^a^	< 0.01
LDL‐C (mmol/L)	3.11 ± 0.97^b^, ^c^	2.97 ± 0.97^a^, ^d^	2.99 ± 0.98^a^	3.06 ± 0.97^b^	< 0.01
HDL‐C (mmol/L)	1.13 ± 0.29^b^, ^c^	1.14 ± 0.30^a^, ^d^	1.17 ± 0.31^a^, ^d^	1.14 ± 0.30^b^, ^c^	< 0.01
TG (mmol/L)	1.93 ± 1.10^b^, ^c^, ^d^	1.82 ± 1.03^a^	1.76 ± 0.96^a^	1.80 ± 0.96^a^	< 0.01

Abbreviations: HDL‐C, high‐density lipoprotein cholesterol; LDL‐C, low‐density lipoprotein cholesterol; TC, total cholesterol; TG, triglycerides.

^a^ *p* < 0.05 versus EDS.

^b^ *p* < 0.05 versus EDS + insomnia.

^c^ *p* < 0.05 versus insomnia.

^d^ *p* < 0.05 versus non‐EDS non‐insomnia.

### 3.4 The Effect of Lipid Lowering Medications, Hypnotics and Diabetes

When patients without lipid lowering medications were analysed separately, the TC differences were not significant. The LDL‐C results were also similar; however, there was a significant difference between patients who experienced both symptoms and those who did not have either EDS or insomnia. The HDL‐C and TG results were similar to the overall group. Additionally, the results were similar to the overall group when patients without hypnotics and nondiabetic subjects were analysed (Table 4).

**TABLE 4:** Comparison of lipid values in patients without lipid‐lowering medications, hypnotics and type II diabetes.

Patients without lipid lowering medications					
	EDS *n* = 2551	EDS + insomnia *n* = 1535	Insomnia *n* = 2112	Non‐EDS non‐insomnia *n* = 3170	*p*
TC (mmol/L)	5.17 ± 1.04	5.10 ± 1.04	5.18 ± 1.09	5.18 ± 1.05	0.20
LDL‐C (mmol/L)	3.18 ± 0.94	3.12 ± 0.92^d^	3.16 ± 0.97	3.22 ± 0.94^b^	0.03
HDL‐C (mmol/L)	1.19 ± 0.34^b^, ^c^	1.22 ± 0.34^a^, ^c^, ^d^	1.28 ± 0.38^a^, ^b^, ^d^	1.20 ± 0.34^b^, ^c^	< 0.01
TG (mmol/L)	1.82 ± 1.01^b^, ^c^, ^d^	1.75 ± 1.00^a^	1.68 ± 0.92^a^, ^d^	1.74 ± 0.94^a^, ^c^	< 0.01

Patients without hypnotics					
	EDS *n* = 3123	EDS + insomnia *n* = 1899	Insomnia *n* = 2527	Non‐EDS non‐insomnia *n* = 4077	*p*
TC (mmol/L)	5.11 ± 1.08^b^, ^c^, ^d^	4.99 ± 1.10^a^	5.05 ± 1.11^a^	5.04 ± 1.10^a^	< 0.01
LDL‐C (mmol/L)	3.12 ± 0.97^b^, ^c^	3.00 ± 0.98^a^, ^d^	3.04 ± 1.00^a^	3.09 ± 0.98^b^	< 0.01
HDL‐C (mmol/L)	1.18 ± 0.33^b^, ^c^	1.21 ± 0.34^a^, ^c^, ^d^	1.27 ± 0.38^a^, ^b^, ^d^	1.20 ± 0.34^b^, ^c^	< 0.01
TG (mmol/L)	1.86 ± 1.04^b^, ^c^, ^d^	1.73 ± 0.95^a^	1.69 ± 0.90^a^, ^d^	1.75 ± 0.93^a^, ^c^	< 0.01

Patients without type II diabetes					
	EDS *n* = 2593	EDS + insomnia *n* = 1672	Insomnia *n* = 2409	Non‐EDS non‐insomnia *n* = 3507	*p*
TC (mmol/L)	5.16 ± 1.06^b^	5.07 ± 1.08^a^	5.12 ± 1.10	5.10 ± 1.08	< 0.01
LDL‐C (mmol/L)	3.17 ± 0.96^b^, ^c^	3.08 ± 0.95^a^, ^d^	3.11 ± 0.98^a^	3.14 ± 0.97^b^	< 0.01
HDL‐C (mmol/L)	1.19 ± 0.33^b^, ^c^	1.23 ± 0.34^a^, ^c^, ^d^	1.28 ± 0.38^a^, ^b^, ^d^	1.21 ± 0.35^b^, ^c^	< 0.01
TG (mmol/L)	1.82 ± 1.02^b^, ^c^, ^d^	1.71 ± 0.90^a^	1.65 ± 0.86^a^, ^d^	1.72 ± 0.93^a^, ^c^	< 0.01

Abbreviations: HDL‐C, high‐density lipoprotein cholesterol; LDL‐C, low‐density lipoprotein cholesterol; TC, total cholesterol; TG, triglycerides.

^a^ *p* < 0.05 versus EDS.

^b^ *p* < 0.05 versus EDS + insomnia.

^c^ *p* < 0.05 versus insomnia.

^d^ *p* < 0.05 versus non‐EDS non‐insomnia.

### 3.5 Comparison of Lipid Values in Patients With Severe OSA

The results were unchanged when only patients with severe OSA were analysed (Table 5).

**TABLE 5:** Comparison of lipid values in patients with severe OSA.

	EDS *n* = 1811	EDS + insomnia *n* = 1212	Insomnia *n* = 1350	Non‐EDS non‐insomnia *n* = 1998	*p*
TC (mmol/L)	5.10 ± 1.09^b^, ^c^	5.00 ± 1.11^a^	4.93 ± 1.09^a^, ^d^	5.02 ± 1.13^c^	< 0.01
LDL‐C (mmol/L)	3.11 ± 0.99^b^, ^c^	3.00 ± 0.99^a^	2.95 ± 0.98^a^, ^d^	3.06 ± 1.01^c^	< 0.01
HDL‐C (mmol/L)	1.14 ± 0.31^b^, ^c^	1.18 ± 0.33^a^, ^d^	1.21 ± 0.33^a^, ^d^	1.16 ± 0.33^b^, ^c^	< 0.01
TG (mmol/L)	1.95 ± 1.08^b^, ^c^, ^d^	1.83 ± 1.02^a^	1.77 ± 0.91^a^	1.83 ± 0.98^a^	< 0.01

Abbreviations: HDL‐C, high‐density lipoprotein cholesterol; LDL‐C, low‐density lipoprotein cholesterol; TC, total cholesterol; TG, triglycerides.

^a^ *p* < 0.05 versus EDS.

^b^ *p* < 0.05 versus EDS + insomnia.

^c^ *p* < 0.05 versus insomnia.

^d^ *p* < 0.05 versus non‐EDS non‐insomnia.

### 3.6 Comparison of Lipid Values in Patients Had Polysomnography as a Diagnostic Test

There was no difference in TC values between the four groups. Otherwise, the results were similar to the overall population (Table 6).

**TABLE 6:** Comparison of lipid values in patients who had polysomnography as a diagnostic test.

	EDS *n* = 2269	EDS + insomnia *n* = 1688	Insomnia *n* = 1978	Non‐EDS non‐insomnia *n* = 2761	*p*
TC (mmol/L)	5.04 ± 1.06	5.00 ± 1.09	4.98 ± 1.11	5.00 ± 1.10	0.07
LDL‐C (mmol/L)	3.04 ± 0.94^c^	3.00 ± 0.96	2.96 ± 0.97^a^	3.03 ± 0.97	0.04
HDL‐C (mmol/L)	1.19 ± 0.34^b^, ^c^	1.21 ± 0.36^a^, ^d^	1.26 ± 0.36^a^, ^d^	1.19 ± 0.35^b^, ^c^	< 0.01
TG (mmol/L)	1.85 ± 1.05^b^, ^c^	1.76 ± 1.00^a^	1.70 ± 0.90^a^, ^d^	1.78 ± 0.96^c^	< 0.01

Abbreviations: HDL‐C, high‐density lipoprotein cholesterol; LDL‐C, low‐density lipoprotein cholesterol; TC, total cholesterol; TG, triglycerides.

^a^ *p* < 0.05 versus EDS.

^b^ *p* < 0.05 versus EDS + insomnia.

^c^ *p* < 0.05 versus insomnia.

^d^ *p* < 0.05 versus non‐EDS non‐insomnia.

### 3.7 Comparison of Lipid Values Based on Different Insomnia Criteria

Shortened sleep duration and prolonged sleep latency were both related to favourable lipid results. In contrast, physician‐based diagnosis of insomnia and hypnotic use were associated with higher TC and TG levels, respectively (Table 7).

**TABLE 7:** Comparison of lipid values based on insomnia criteria.

Duration of sleep time			
	Short sleep time (*n* = 2188)	Normal sleep time (*n* = 9965)	*p*
TC (mmol/L)	4.98 ± 1.10	5.07 ± 1.10	< 0.01
LDL‐C (mmol/L)	3.01 ± 0.97	3.08 ± 0.99	< 0.01
HDL‐C (mmol/L)	1.22 ± 0.35	1.21 ± 0.35	< 0.01
TG (mmol/L)	1.69 ± 0.89	1.78 ± 0.98	< 0.01

Sleep latency			
	Prolonged sleep latency (*n* = 3637)	Normal sleep latency (*n* = 8516)	*p*
TC (mmol/L)	5.03 ± 1.11	5.06 ± 1.10	0.02
LDL‐C (mmol/L)	3.02 ± 0.99	3.09 ± 0.98	< 0.01
HDL‐C (mmol/L)	1.25 ± 0.37	1.19 ± 0.34	< 0.01
TG (mmol/L)	1.72 ± 0.93	1.79 ± 0.97	< 0.01

Physician diagnosed insomnia			
	Yes (*n* = 388)	No (*n* = 11,521)	*p*
TC (mmol/L)	5.21 ± 1.10	5.05 ± 1.10	0.02
LDL‐C (mmol/L)	3.19 ± 0.99	3.07 ± 0.99	0.09
HDL‐C (mmol/L)	1.25 ± 0.38	1.21 ± 0.35	0.26
TG (mmol/L)	1.81 ± 1.00	1.77 ± 0.96	0.13

Hypnotics			
	On hypnotics (*n* = 527)	Off hypnotics (*n* = 11,626)	*p*
TC (mmol/L)	5.00 ± 1.20	5.05 ± 1.10	0.83
LDL‐C (mmol/L)	2.96 ± 0.97	3.07 ± 0.98	0.18
HDL‐C (mmol/L)	1.23 ± 0.37	1.21 ± 0.35	0.09
TG (mmol/L)	1.84 ± 1.02	1.76 ± 0.96	< 0.01

Abbreviations: HDL‐C, high‐density lipoprotein cholesterol; LDL‐C, low‐density lipoprotein cholesterol; TC, total cholesterol; TG, triglycerides.

## 4 Discussion

Analysing a large cohort of patients with OSA we reported a significant relationship between EDS and dyslipidaemia. The association was present in most subgroups, but not when females were analysed separately. While the prevalence of CVD was higher in insomnia, this phenotype was associated with more favourable lipid values. However, this needs to be interpreted with caution, as in the subgroup of patients who were diagnosed with chronic insomnia by a physician and in those who took hypnotics, the lipid values were deranged.

A significant dose‐dependent relationship was reported between OSA severity and dyslipidaemia in a large group of patients participating in the ESADA cohort (Gunduz et al. 2019; Gündüz et al. 2018). In addition, CPAP treatment was associated with effective reduction in serum lipid levels (Gunduz et al. 2020). However, the presence of EDS and insomnia has not been explored in the previous reports investigating lipid levels in ESADA (Gunduz et al. 2019, 2020; Gündüz et al. 2018). Insomnia is particularly important, as it was associated with worse adherence to CPAP in ESADA (Saaresranta et al. 2016) and other cohorts (Björnsdóttir et al. 2013; Pieh et al. 2013). Contrarily, patients reporting EDS are more likely to use their CPAP (Saaresranta et al. 2016); therefore, a CPAP‐related improvement in dyslipidaemia is more expected in these patients. However, pre‐CPAP sleepiness was not related to the efficacy of CPAP on lipid profiles according to a meta‐analysis analyzing 14 randomised controlled trials (Chen et al. 2022).

EDS was associated with deranged lipid values in both the whole cohort and subanalyses. There are multiple potential reasons that need to be investigated in dedicated studies. First, EDS is often associated with sedentary behaviour and increased dietary intake, especially in the form of fat and carbohydrates (Sánchez‐de‐la‐Torre et al. 2011). In line with this, the BMI in the EDS groups was higher in the current study. While, the lipid results were adjusted for BMI, no data on dietary intake was available in ESADA. Second, the prevalence of type II diabetes was higher in the EDS group confirming the previous results (Chen et al. 2024). Insulin resistance is one of the most recognised risk factors for dyslipidaemia, particularly hypertriglyceridaemia, in OSA (Meszaros and Bikov 2022). Third, patients in the EDS groups had more severe OSA. As discussed before, OSA severity is related to a worse lipid profile (Gunduz et al. 2019; Gündüz et al. 2018). Of note, analyses were adjusted for AHI and T90, and subgroup analyses have been performed in severe OSA separately. Fourth, EDS could be a consequence of an altered gut microbiome due to some gamma‐aminobutyric acid‐producing bacteria, that can also lead to cardiometabolic alterations in OSA (Bikov et al. 2022).

Patients with OSA complaining about insomnia symptoms had a higher prevalence of CVD. This association is particularly remarkable considering that the insomnia groups comprised more females than the two non‐insomnia groups. However, it is noteworthy that a significant proportion of women in the insomnia groups were likely postmenopausal based on the average age of the cohort. Menopause is associated with CVD (Yoshida et al. 2021), central obesity and OSA (Wang et al. 2025). Unfortunately, data on menopause was not collected in ESADA. Another possible explanation for the higher CVD prevalence could be the higher frequency of hypertension in the insomnia group. Hypertension is a known consequence of chronic insomnia, especially in those with short sleep duration (Fernandez‐Mendoza et al. 2019; Vgontzas et al. 2009, 2013), and patients with OSA suffering from insomnia symptoms had more prevalent hypertension in the SHHS (Lechat et al. 2022b). Considering the lower prevalence of dyslipidaemia in patients reporting insomnia symptoms in ESADA, the association between insomnia and CVD is likely driven by hypertension.

It is worth noting that insomnia was associated with lower LDL‐C, TG and higher HDL‐C in the whole population. These results are contrasting to the findings of population‐based studies in chronic insomnia (Lee et al. 2024; Syauqy et al. 2019; Wang et al. 2023). Of note, these studies were not conducted in patients with OSA. Intersex differences in the prevalence of insomnia and lipid values could explain some discrepancies. Indeed, Silva‐Costa et al. reported a significant association between insomnia and higher TG values only in males (Silva‐Costa et al. 2020). In line, there were no differences in TG and TC values between the groups in women in our study. In contrast, female patients with insomnia had still higher HDL‐C and lower LDL‐C. Patients with insomnia were more likely to be taking lipid‐lowering medications. However, even when these patients were excluded, HDL‐C values were higher and TG levels were lower in the insomnia group. The somewhat unexpected results could be explained by the insomnia definition used in this study. Both shorter sleep time and prolonged sleep latency were associated with favourable lipid values. In contrast, physician‐based diagnosis of insomnia as well as hypnotic use were related to higher TC and TG levels. This prompts using standardised insomnia criteria (such as defined by the International Classification of Sleep Disorders) rather than insomnia symptoms in further studies. Indeed, comorbid insomnia and sleep apnoea (COMISA) were associated with metabolic syndrome in various population‐based studies (Solelhac et al. 2025).

The strengths of the study, including the large sample size and diversity of participants allowed robust subgroup analyses. Apart from the insomnia definition, discussed before, the study has further limitations. First, it had a cross‐sectional nature. Analysis of posttreatment lipid values, especially in the EDS group could better define the relationship between EDS and dyslipidaemia and could provide causality. Second, polysomnography was performed only in two thirds of patients. While the results were similar when only those who had polysomnography as a diagnostic test were analysed, further subgroup analyses based on the objective total sleep time could better delineate the relationship between insomnia and lipid values (Fernandez‐Mendoza et al. 2017). Third, novel physiological markers, such as the hypoxic burden or heart rate variability could give us a better understanding of the observed results. Unfortunately, these were not available in ESADA. Fourth, the severity of insomnia was not quantified using standardised questionnaires limiting the comparability of our results. Finally, we did not record lifestyle factors, such as diet and physical exercise that could affect lipid values.

## 5 Conclusion

EDS, but not insomnia, was associated with dyslipidaemia in patients with OSA. The study highlights that phenotyping based on symptoms may be useful in detecting cardiovascular and metabolic diseases, and insomnia and daytime sleepiness may relate to different cardiometabolic pathways.

## Author Contributions

A.B. and S.M. developed the hypothesis and analysis plan, and drafted the manuscript. A.B. performed statistical analysis. S.B., U.A., T.S., O.K.B., S.S., I.B., P.S., A.P., D.T., F.F., H.G., L.G., S.M. participated in recruitment and critically reviewed the manuscript.

## Conflicts of Interest

Andras Bikov declares speaker fees from Idorsia, Astra Zeneca, Berlin‐Chemie Menarini and Inspire Medical Systems. Ludger Grote reports lecturing activities for Resmed, Philips, Astra Zeneca, Itamar and Lundbeck as well as grant support for scientific projects from Desitin and Bayer. He has a co‐ownership in a licensed patent for sleep apnea treatment. Tarja Saaresranta declares speaker fees from ResMed, Finnish Medical Association Duodecim, Idorsia and Boehringer Ingelheim. Dries Testelmans declares speaker fees from ResMed and Philips Respironics. Ulla Anttalainen declares speaker fees from ResMed, Finnish Medical Association Duodecim, Boehringer‐Ingelheim and Fisher & Paykel. The other authors declare no conflicts of interest.

## Data Availability

The data that support the findings of this study are available on request from the corresponding author. The data are not publicly available due to privacy or ethical restrictions.

## Appendix A ESADA Collaborators: Group Authorship

Steiropoulos P.^1^; Verbraecken J.^2^; Petiet E.^2^; Georgia Trakada^3^; Fietze I.^4^; Penzel T.^4^; Ondrej Ludka^5^; Bouloukaki I.^6^; Schiza S.^6^; McNicholas W. T.^7^; Ryan S.^8^; Riha R. L.^9^; Kvamme J. A.^10^; Grote L.^11,12^; Hedner J.^11,12^; Zou D.^11,12^; Katrien Hertegonne^13,14^; Dirk Pevernagie^13,14^; Bailly S.^15,16^; Pépin J. L.^15,16^; Tamisier R.^15,16^; Hein H.^17^; Basoglu O. K.^18^; Tasbakan M. S.^18^; Joppa P.^19,20^; Staats R.^21^; Dries Testelmans^22^; Alexandros Kalkanis^23^; Haralampos Gouveris^24^; Ludwig K.^24^; Lombardi C.^25,26^; Parati G.^25,26^; Bonsignore M. R.^27^; Francesco Fanfulla^28^; Petitjean M.^29^; Roisman G.^29^; Drummond M.^30^; van Zeller M.^30^; Randerath W.^31^; Mathes S.^31^; Dogas Z.^32^; Galic T.^32^; Pataka A.^33^; Mihaicuta S.^34^; Anttalainen U.^35,36^; Saaresranta T.^35,36^; Sliwinski P.^37^

^1^Sleep Unit, Department of Pneumonology, Democritus University of Thrace, Alexandroupolis, Greece

^2^Multidisciplinary Sleep Disorders Centre, Antwerp University Hospital and University of Antwerp, Antwerp, Belgium

^3^Pulmonary Medicine, National and Kapodistrian University of Athens, Athens, Greece

^4^Schlafmedizinisches Zentrum, Charité—Universitätsmedizin Berlin, Germany

^5^Department of Cardiology, University Hospital Brno and International Clinical Research Center, St. Ann's University Hospital, Brno, Czech Republic

^6^Sleep Disorders Unit, Department of Respiratory Medicine, Medical School, University of Crete, Greece

^7^Department of Respiratory Medicine, St. Vincent's University Hospital, Dublin, Ireland

^8^Pulmonary and Sleep Disorders Unit, St. Vincent's University Hospital, Dublin, Ireland

^9^Department of Sleep Medicine, Royal Infirmary Edinburgh, Scotland

^10^Sleep Laboratory, ENT Department, Førde Central Hospital, Førde, Norway

^11^Sleep Disorders Center, Pulmonary Department, Sahlgrenska University Hospital, Göteborg, Sweden

^12^Center of Sleep and Wake Disorders, Sahlgrenska Academy, Gothenburg University, Göteborg, Sweden

^13^Department of Respiratory Medicine, Ghent University Hospital, Gent, Belgium

^14^Department of Internal Medicine and Pediatrics, Faculty of Medicine and Health Sciences, Ghent University, Gent, Belgium

^15^Université Grenoble Alpes, INSERM HP2 (U1042)

^16^Grenoble University Hospital, Grenoble, France

^17^Sleep Disorders Center, St. Adolf Stift, Reinbeck, Germany

^18^Department of Chest Diseases, Ege University, Izmir, Turkey

^19^Department of Respiratory Medicine and Tuberculosis, Faculty of Medicine, P.J. Safarik University, Kosice, Slovakia

^20^L. Pasteur University Hospital, Kosice, Slovakia

^21^Department of Respiratory Medicine, Hospital de Santa Maria, Lisbon, Portugal

^22^Sleep Disorders Centre, University Hospital Gasthuisberg, Leuven, Belgium

^23^Department of Respiratory Diseases, Louvain University Center for Sleep and Wake Disorders (LUCS), University Hospitals Leuven, KU Leuven, Leuven, Belgium

^24^ENT Department at Mainz University Hospital, Mainz, Germany

^25^Istituto Auxologico Italiano, IRCCS, Department of Cardiovascular, Neural and Metabolic Sciences, St. Luke Hospital, Milan, Italy

^26^Department of Medicine and Surgery, University of Milano‐Bicocca, Milan, Italy

^27^PROMISE Department, University of Palermo, Palermo, Italy

^28^Unità Operativa di Medicina del Sonno, Istituto Scientifico di Pavia IRCCS, Pavia, Italy

^29^Unité de Médecine du Sommeil, Hopital Antoine‐Beclere, Clamart, France

^30^Pulmonology Department Hospital São João, Medicine Faculty of Porto University, Porto, Portugal

^31^Sleep Disorders Centre, Pulmonary Clinic, Solingen, Germany

^32^Sleep Medicine Center, Department of Neuroscience, University of Split School of Medicine, Split, Croatia

^33^Respiratory Failure Unit, G. Papanikolaou Hospital, Thessalonika, Greece

^34^Pulmonary Department, Victor Babes University of Medicine and Pharmacy, Victor Babes Hospital, Timisoara, Rumania

^35^Division of Medicine, Department of Pulmonary Diseases, Turku University Hospital, Turku, Finland

^36^Sleep Research Centre, Department of Pulmonary Diseases and Clinical Allergology, University of Turku, Turku, Finland

^37^Second Department of Respiratory Medicine, Institute of Tuberculosis and Lung Diseases, Warsaw, Poland
